# Investigation of the Effects of Internal and External Lateral Osteotomies on Periorbital Edema and Ecchymosis in Patients Undergoing a Septorhinoplasty

**DOI:** 10.7759/cureus.9609

**Published:** 2020-08-07

**Authors:** Ergin Bilgin, Deniz Baklacı

**Affiliations:** 1 Otolaryngology - Head and Neck Surgery, Bülent Ecevit University Hospital, Zonguldak, TUR; 2 Otolaryngology, Bülent Ecevit University Faculty of Medicine, Zonguldak, TUR

**Keywords:** lateral osteotomy, external, internal, edema, ecchymosis, septorhinoplasty

## Abstract

Objective: Periorbital ecchymosis and edema are common after septorhinoplasty surgery. This study aimed to compare internal and external lateral nasal osteotomies performed in septorhinoplasty in terms of postoperative ecchymosis and edema.

Methods: Patients who underwent septorhinoplasty between January 2020 and July 2020 in our clinic were included in the study. In all patients, right lateral nasal osteotomies were performed endonasally and left lateral nasal osteotomies externally. The postoperative 1st, 7th, and 14th day ecchymosis and edema scores of all patients were calculated separately for the two groups and compared.

Results: A total of 60 patients (29 females, 31 males) were included in the study. The mean age of the patients was 33.88 ± 10.30 years. No significant difference was observed between the two groups in terms of the postoperative periorbital ecchymosis scores on the first day and the first and second weeks (0.314, 0.344, and 0.468, respectively). There was also no significant difference between the two groups in terms of the postoperative periorbital edema scores on the first day and at the first and second weeks (0.272, 0.359, and 0.513, respectively).

Conclusion: The results obtained from this study showed no significant difference in the periorbital ecchymosis and edema scores between the patients who had undergone septorhinoplasty with internal or external lateral osteotomies. Further multicenter studies are recommended to verify the findings of this study with a larger sample size.

## Introduction

Septorhinoplasty is a common surgical procedure. Unfortunately, as in all surgical procedures, there are well-known risks, such as intraoperative bleeding, postoperative pain, periorbital edema, and ecchymosis depending on the surgical procedures performed during septorhinoplasty [1]. The degree of periorbital ecchymosis and edema can range from a slight color change to a severe form that disrupts the patient’s vision [2,3]. Patients often have questions concerning the new appearance of their nose after surgery, as well as whether there will be swelling around the eyes or the degree of swelling after esthetic nasal surgery. Surgeons try to respond to these questions by focusing on the surgical procedures and medical materials to be used in an attempt to minimize the anxiety of patients.

After rhinoplasty, ecchymosis and edema may occur at different degrees depending on some preventable/changeable factors, such as coagulation disorders, excess subcutaneous adipose tissue removal, type of surgical procedure, osteotomy type, length of surgery, localization differences of the angular artery, and presurgical drug use [4,5].

A lateral osteotomy is one of the main components of septorhinoplasty operations performed for the purpose of reconstructing the nasal pyramid [6]. In lateral osteotomies, the angular artery and periosteum are usually damaged, which is considered to be the main cause of postoperative edema and periorbital ecchymosis [7]. On the other hand, in many cases, this step is essential for narrowing the nasal pyramid [4,8,9]. A lateral osteotomy can be performed by percutaneous or endonasal methods [10].

Various strategies have been applied to reduce periorbital edema and ecchymosis, including the use of steroids during and after surgery, intraoperative hypotension, postoperative cold compress, use of subperiosteal osteotomy techniques, applying spot pressure on the damaged area, and plaster applications [2,6,11-19]. Single-dose dexamethasone has also been described to reduce postoperative edema and ecchymosis. However, lidocaine and epinephrine (1: 100,000) injections have been reported to have no significant effect before lateral osteotomies [14].

A few corticosteroid injections at high doses have also been shown to reduce postoperative edema and ecchymosis [15]. It has been proven that the application of cold compress around the periorbital region and nose area alleviates periorbital edema and ecchymosis [16]. The method to be applied should not only be effective in reducing such adverse effects, but it should also be simple, feasible, and cost-effective with little or no side effects. Despite all the proposed solutions, none is able to fully prevent complications related to the surgical procedure, and therefore periorbital edema and ecchymosis remain to be the main concerns after septorhinoplasty.

In some studies, an endonasal lateral osteotomy has been shown to result in less periorbital edema and ecchymosis compared to the external technique [17,20,21]. Meymaneh et al. reported that an external lateral osteotomy was superior to the endonasal technique in terms of reducing periorbital edema and ecchymosis [22]. Considering the controversial results regarding this issue, the current study was conducted to evaluate the effects of endonasal and external lateral nasal osteotomies on the development of periorbital edema and ecchymosis.

## Materials and methods

This retrospective study included 60 patients who underwent septorhinoplasty between January 2020 and July 2020 in our clinic and had regularly/updated records available. All operations were performed by the same surgeon (B.E.). In all patients, right lateral nasal osteotomies were performed endonasally and left lateral nasal osteotomies externally. Patients who underwent resection due to a hump (of cartilage or bone origin) in the nasal dorsum and those who had not previously undergone any sinonasal surgery were included. Septorhinoplasty was performed in all patients. Coagulation parameters were within normal limits in all patients, and there was no history of alcohol, drug use, or smoking. Hypotensive anesthesia was induced with remifentanil. Ecchymosis and edema scores were calculated on the 1st, 7th, and 14th postoperative days using photographs taken with the permission of the patients. Ecchymosis and edema scoring was undertaken using the staging system previously described in the literature [23].

Surgical technique

Skin flaps were prepared using a V-shaped columellar skin incision. The periosteum of the nasal bone was lifted using a periosteal elevator, followed by the separation of the upper lateral cartilage from the septum using scissors. Then, the cartilage dorsum was removed using scissors and the bone dorsum was removed using a 6-mm osteotome with protector. A spreader or autospreader flap was used to prevent inverted-V deformity. In the last stage of the operation, high-low-high internal and external lateral osteotomies were performed. Lateral nasal osteotomies were performed endonasally on the right with a 3-mm curved osteotome with protector and externally on the left using a 3-mm osteotome without protector. Periosteal elevation was not undertaken before lateral nasal osteotomies since they were considered to increase edema and ecchymosis. At the end of surgery, columellar skin was sutured using 5/0 prolene sutures.

Statistical analysis

Mean and standard deviation values were used in descriptive statistics concerning continuous data, and the Shapiro-Wilk test was conducted to analyze the compliance of continuous data to normal distribution. The independent samples t-test was used to compare the postoperative ecchymosis and edema scores on the first day and at the first and second weeks. IBM SPSS Statistics v. 20 (Armonk, NY: IBM Corp.) was used for the statistical evaluations, and p < 0.05 was accepted as the statistical significance limit.

## Results

A total of patients were included in the study. The mean age of the patients was 33.88 ± 10.30 years (Table 1).

**Table 1:** Demographic characteristics of the patients SD: standard deviation

Characteristics	Values
Age (mean ± SD)	33.88 ± 10.30
Gender, n (%)	
Female	29 (48.4)
Male	31 (51.6)

The postoperative first-day and first- and second-week periorbital ecchymosis scores were 2.75 ± 0.70, 1.38 ± 0.58, and 1.05 ± 0.2, respectively for the external osteotomy group and 2.88 ± 0.73, 1.48 ± 0.56, and 1.08 ± 0.27, respectively for the internal osteotomy group. There was no significant difference between the two groups in terms of the postoperative periorbital ecchymosis scores at any measurement time (p = 0.314, 0.344, and 0.468 for the first day, first week, and second week, respectively) (Table 2).

**Table 2:** Ecchymosis scores of the groups SD: standard deviation

	Score (mean ± SD)		
	External group	Internal group	P values
Day 1	2.75 ± 0.70	2.88 ± 0.73	0.314
Week 1	1.38 ± 0.58	1.48 ± 0.56	0.344
Week 2	1.05 ± 0.21	1.08 ± 0.27	0.468

The mean postoperative periorbital edema scores on the first day and at the first and second weeks were 2.76 ± 0.69, 1.43 ± 0.62, and 1.06 ± 0.25, respectively, for the external osteotomy group and 2.91 ± 0.78, 1.53 ± 0.56, and 1.10 ± 0.30, respectively, for the internal osteotomy group. There was no significant difference between the two groups in terms of periorbital edema for any evaluation time (p = 0.272, 0.359, and 0.513 for the first day, first week, and second week, respectively) (Table 3).

**Table 3:** Edema scores of the groups SD: standard deviation

	Score (mean ± SD)		
	External group	Internal group	P values
Day 1	2.76 ± 0.69	2.91 ± 0.78	0.272
Week 1	1.43 ± 0.62	1.53 ± 0.56	0.359
Week 2	1.06 ± 0.25	1.10 ± 0.30	0.513

## Discussion

The data obtained from this study revealed no statistically significant difference between the two groups in terms of the postoperative periorbital ecchymosis and edema scores. Motamed et al. compared internal and external lateral nasal osteotomies in relation to the development of periorbital edema and ecchymosis [24]. The authors evaluated 168 patients, who had undergone rhinoplasty, on the postoperative 1st, 3rd, 7th, and 30th days, and reported no significant difference between the two methods, which is consistent with our research. Similarly, van Loon used with three-dimensional stereophotogrammetry to evaluate patients for periorbital edema after internal and external lateral nasal osteotomies and found no significant difference between the two methods [25]. In another study including 20 patients undergoing rhinoplasty with external and internal lateral nasal osteotomies, Yücel reported the similar periorbital edema scores on the second and seventh days [26].

Periorbital edema and ecchymosis affect the outcomes of rhinoplasty and cause concerns and dissatisfaction among patients, their relatives, and surgeons. Various methods have been proposed for lateral nasal osteotomies in order to reduce postoperative complications. However, surgeons have different views concerning the best practice. Some researchers, such as Meymaneh et al. and Ford et al. are in favor of using the external technique, suggesting that this method reduces trauma to the soft tissue, nasal mucosa, and periosteum [24,27].

Yazdani et al. evaluated nasal mucosal rupture damage and periorbital edema and ecchymosis associated with the internal and external lateral nasal osteotomy techniques [28]. The results showed that there was less nasal mucosal rupture damage and lower periorbital edema and ecchymosis scores in the external group than in the internal group; however, this decrease was not statistically significant. In a study conducted by Giacomarra et al., the authors stated that they preferred the external method over the internal method since it led to less mucosal damage [9].

Yücel showed that the ecchymosis score was significantly higher in the internal lateral nasal osteotomy group compared to the group in which the external technique was applied; however, he observed no statistically significant difference in the ecchymosis scores of the two groups on the postoperative seventh day [26]. In a 30-case series, Hashemi et al. reported that the patients who had been operated with the external method had decreased postoperative first-day edema and ecchymosis scores [29]. In the same study, the severity of ecchymosis was found to be significantly lower at the end of the postoperative seventh day in the external osteotomy group. In contrast, Denneny and Tardy, who investigated the internal method using 2-3 mm osteotomes, reported that the internal method reduced edema, ecchymosis and mucosal damage [30].

There are many studies reporting contradicting results; therefore, there is still no consensus among surgeons for the ideal lateral nasal osteotomy method. In general, researchers advocating the external method believe that it protects the periosteum, provides better control over bone fractures, and prevents nose collapse and intranasal problems. In addition, this method results in less bleeding, ecchymosis and edema rates since it causes less tissue trauma. On the other hand, those suggesting the use of the internal osteotomy method consider that when this method is applied with high precision, it leads to lower rates of edema and ecchymosis.

Another factor to be taken into consideration in the choice of the osteotomy method is the surgeon’s ability or experience related to rhinoplasty, which can affect surgical outcomes. In this context, the lateral nasal osteotomy method depends more on the experience of the surgeon rather than his/her scientific knowledge.

## Conclusions

The results of this study showed no significant difference in the postoperative periorbital ecchymosis and edema scores of the patients who underwent internal and external lateral osteotomies during septorhinoplasty; therefore, we recommend that surgeons choose the optimal method based on its ease of implementation and lower complexity in line with their experience and discretion. Further multicenter studies are warranted to verify the findings of the current study in a larger sample size.
